# Two Cases of Ischemic Complications in Abdominoplasty After Use of a New Biologic Migraine Medication

**DOI:** 10.1097/GOX.0000000000006449

**Published:** 2025-01-14

**Authors:** Reetta Tuominen, Virve Koljonen

**Affiliations:** From the * Department of Plastic Surgery, Helsinki University Hospital and University of Helsinki, Helsinki, Finland; † Eira Hospital, Helsinki, Finland.

## Abstract

Abdominoplasty is a common aesthetic procedure, and ischemic complications are rare, particularly in nonsmokers. We present 2 cases of ischemic complications in nonsmoking patients treated with fremanezumab, a biologic medication for severe migraines. A 55-year-old woman underwent lipoabdominoplasty on December 18, 2023. At the 16-day postoperative follow-up, demarcated necrosis was observed beneath the wound tape, and secondary direct closure was performed 6 weeks later. She had been using fremanezumab for 4 years. A 47-year-old woman underwent abdominoplasty on April 23, 2024, with moderate dissection and liposuction to the flanks. Signs of abdominal flap ischemia were evident in the operating room, and treatment for the Raynaud phenomenon was initiated immediately. The ischemia demarcated over 2 weeks, and secondary direct closure was performed 3 weeks postoperatively. She had been using fremanezumab for two months. Calcitonin gene-related peptide antagonists are potent medications for severe migraine with few contraindications. Fremanezumab may affect peripheral circulation, potentially increasing the risk of surgical complications.

Abdominoplasty is generally considered a safe surgery, typically associated with only minor complications and no long-term sequelae. Severe complications are infrequent.^1^

Blocking calcitonin gene-related peptide (CGRP) has emerged as a potential mechanism for preventing migraine attacks.^2^ Two classes of drugs block CGRP: CGRP receptor antagonists and monoclonal antibodies targeting either CGRP or its receptor.^2^ CGRP antagonists are used as a third-line treatment to prevent migraines in adults who experience them at least 4 days per month.^3,4^ Fremanezumab is a monoclonal antibody administered as a monthly subcutaneous injection.

CGRP antagonists are considered safe.^2^ According to the European Medicines Agency package leaflet, adverse reactions to Ajovy (fremanezumab) include local reactions at the injection site (eg, pain, induration, erythema) in more than 10% of cases, as well as hypersensitivity reactions such as rash, pruritus, urticaria, and, rarely, anaphylaxis.^5^

The literature reports an association between CGRP antagonists and a noxious impact on the vasodilatory response, particularly in patients with the Raynaud phenomenon (RP).^6^ RP is characterized by vasospasm, causing affected areas to turn white (pallor) due to reduced blood flow during cold exposure or stress. These areas subsequently turn blue from deoxygenation and then red due to reperfusion, which can cause numbness and pain. RP is diagnosed clinically and can be primary or secondary, often associated with connective tissue diseases such as scleroderma, malignancy, or atherosclerosis.

## PRESENTATION OF CASES

### Case 1

A 55-year-old woman (body mass index 21.6 kg/m^2^) experienced a pulmonary embolism in 2022 and used apixaban for 12 months. Genetic testing ruled out predisposition to blood clotting disorders. Being a never-smoker, she had not undergone any abdominal wall operations or treatments. Her medical history included a symptomatic cesarean section scar fixed to the fascia, a small umbilical hernia, and mild rectus diastasis.

On December 8, she received the monthly subcutaneous injection of fremanezumab in the abdomen. The patient underwent the hydrodissection-assisted extended lateral plication (HELP)–abdominoplasty modification^7^ on December 18, 2023. At the 16-day postoperative follow-up, she presented with demarcated necrosis under the wound tape. The necrosis was revised bedside, and negative-pressure wound therapy was initiated, but no progression was noted after 2 weeks. Revision surgery was scheduled 5 weeks and 6 days postoperatively. Minor residual fat necrosis below Scarpa’s fascia was removed, and after mobilization of the supraumbilical flap, the wound was closed directly.

After case 2, a review of case 1’s medical records highlighted the use of fremanezumab for 4 years. Retrospectively, the patient reported always having cold peripheries, unaffected by fremanezumab.

### Case 2

A 47-year-old woman (body mass index 22.5 kg/m²) was using progesterone-only birth control pills, methylphenidate, citalopram, and bisoprolol for arrhythmia. She had briefly used tobacco products in her youth and had not undergone any previous abdominal wall operations or treatments. The patient lost 20 kg with the assistance of ongoing semaglutide treatment and presented with excess skin and moderate, postpregnancy symptomatic rectus diastasis with an umbilical hernia. A lipoabdominoplasty with rectus diastasis repair and umbilical hernia correction was scheduled on April 23, 2024, using the HELP technique.

**Fig. 1.**
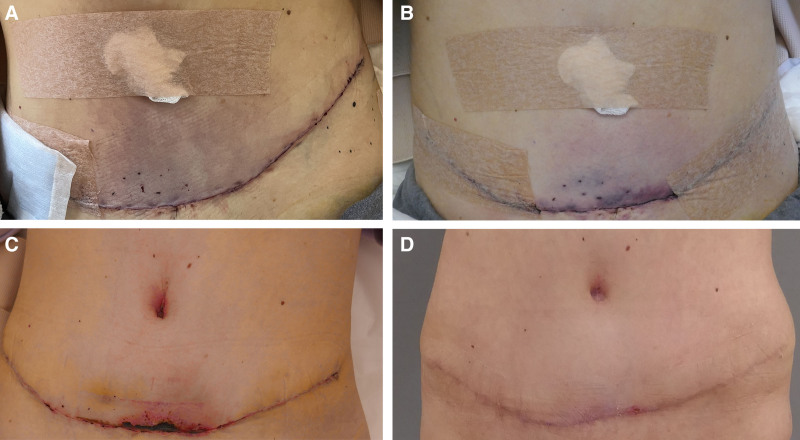
Postoperative results over time. A, A photograph of the abdomen taken 2 hours postoperatively, showing ischemia affecting approximately 1% of the total body surface area of the abdominal flap. B, A photograph of the abdomen taken on the first postoperative day, after the initiation of Raynaud medication, showing a decreasing ischemic area. C, A photograph showing necrosis demarcation after 2 weeks. D, A photograph taken 6 weeks after secondary closure.

During the procedure, 400 mL of water-assisted liposuction was performed on the flanks, with no liposuction conducted medially. The abdominal flap was dissected at the fascia layer using electrocautery, preserving as many lateral vessels as possible. No sharp instruments were used in manipulating the flap. The diastasis width was 3 cm, the hernia defect was 8 mm, and the total plicated fascia width was 8 cm. The operation lasted four hours. During closure, the abdominal skin appeared bluish, and an ischemic area covering approximately 1% of the total body surface area on the abdominal flap was noted in the recovery room (Fig. 1A).

The patient reported that her 2 most recent fremanezumab injections, the latest administered 3 weeks preoperatively, caused cold, pale digits as a side effect, requiring her to warm her feet and hands. A focused literature review indicated that fremanezumab might exacerbate RP. Empirical treatment for RP was initiated 4 hours postoperatively, consisting of amlodipine 10 mg administered twice daily and isosorbide mononitrate 10 mg once daily. Topical calcium channel blockers were unavailable. Additionally, the abdominal binder was removed, warm blankets were applied, and cephalexin along with enoxaparin 40 mg were prescribed. The ischemic area decreased by the first postoperative day (Fig. 1B), and demarcation progressed for 2 weeks (Fig. 1C). Amlodipine and isosorbide mononitrate were continued for 2 weeks. Three weeks postoperatively, skin necrosis had reduced to 0.5 × 3 cm, but sub-Scarpa’s fascia fat necrosis warranted surgical intervention. During secondary closure, approximately 50 mL of nonvital fat tissue was removed. The abdomen 6 weeks after secondary closure is shown in Figure 1D.

## DISCUSSION

We report 2 cases of ischemic complications in nonsmokers using fremanezumab for migraine. Skin necrosis due to insufficient perfusion of the abdominal flap occurs in 3%–4.4% of the cases.^8^ The reoperation rate for severe ischemia is less than 1%.^8^

In 2023, the corresponding author performed 35 HELP-abdominoplasties at Eira Hospital with the same team (19 without liposuction and 14 with liposuction). Case 1 was the second Clavien-Dindo grade III complication; the first was a hematoma, resulting in a necrosis rate of 2.8%. In 2024, the same team performed 34 abdominoplasties (23 without liposuction and 11 with liposuction), with case 2 being the only Clavien-Dindo grade III complication, resulting in a necrosis rate of 2.9%. Both patients exhibited Raynaud-type symptoms: case 1 before using fremanezumab and case 2 afterward.

Migraine has a global prevalence of 14%–15%, accounting for 4.9% of worldwide ill health, as measured in years lived with disability.^4^ Migraine often occurs simultaneously with RP.^9^

In the study by Breen et al,^6^ 5.3% of RP patients using CGRP antagonists for migraine experienced microvascular complications, ranging from worsening RP to gangrene and autonecrosis, requiring distal digit amputation. Evans^10^ also reported 2 cases where use of CGRP antagonists was associated with digital ulceration in patients with underlying RP.

CGRP antagonists are projected to dominate the migraine medication market by 2026. The combination of migraine, RP, and CGRP antagonist use is expected to become increasingly common.

## CONCLUSIONS

Precautions may be necessary when planning surgery for patients taking CGRP antagonist medications, especially those with RP. It is crucial to inform patients about the potential impact of CGRP antagonists on surgical outcomes.

## DISCLOSURE

The authors have no financial interest to declare in relation to the content of this article.

## References

[1] Staalesen T Elander A Strandell A. A systematic review of outcomes of abdominoplasty. J Plast Surg Hand Surg. 2012;46:139–144. 22747350 10.3109/2000656X.2012.683794

[2] Deen M Correnti E Kamm K; European Headache Federation School of Advanced Studies (EHF-SAS). Blocking CGRP in migraine patients—a review of pros and cons. J Headache Pain. 2017;18:96. 28948500 10.1186/s10194-017-0807-1 PMC5612904

[3] Eigenbrodt AK Ashina H Khan S. Diagnosis and management of migraine in ten steps. Nat Rev Neurol. 2021;17:501–514. 34145431 10.1038/s41582-021-00509-5 PMC8321897

[4] Ashina M Terwindt GM Al-Karagholi MAM. Migraine: disease characterisation, biomarkers, and precision medicine. Lancet (London, England). 2021;397:1496–1504. 33773610 10.1016/S0140-6736(20)32162-0

[5] Ajovy. Available at www.ema.europa.eu/en/documents/product-information/ajovy-epar-product-information_en.pdf. Accessed April 23, 2024.

[6] Breen ID Brumfiel CM Patel MH. Evaluation of the safety of calcitonin gene-related peptide antagonists for migraine treatment among adults With Raynaud phenomenon. JAMA Netw Open. 2021;4:e217934. 33871613 10.1001/jamanetworkopen.2021.7934 PMC8056280

[7] Tuominen R Peltoniemi H Jahkola T. An abdominoplasty modification for post-pregnancy abdomen with rectus diastasis and midline hernia: the technique and results. Plast Reconstr Surg. 2023;153:1111e–1115e. 10.1097/PRS.0000000000010637 PMC11350177 37192371

[8] Vidal P Berner JE Will PA. Managing complications in abdominoplasty: a literature review. Arch Plast Surg. 2017;44:457–468. 28946731 10.5999/aps.2017.44.5.457 PMC5621815

[9] Zahavi I Chagnac A Hering R. Prevalence of Raynaud’s phenomenon in patients with migraine. Arch Intern Med. 1984;144:742–744. 6143540

[10] Evans RW. Raynaud’s phenomenon associated with calcitonin gene-related peptide monoclonal antibody antagonists. Headache. 2019;59:1360–1364. 31310337 10.1111/head.13596

